# Association between Polymorphisms in the Fatty Acid Desaturase Gene Cluster and the Plasma Triacylglycerol Response to an *n*-3 PUFA Supplementation

**DOI:** 10.3390/nu4081026

**Published:** 2012-08-17

**Authors:** Hubert Cormier, Iwona Rudkowska, Ann-Marie Paradis, Elisabeth Thifault, Véronique Garneau, Simone Lemieux, Patrick Couture, Marie-Claude Vohl

**Affiliations:** 1 Institute of Nutraceuticals and Functional Foods (INAF), Laval University, Quebec G1V 0A6, Canada; Email: iwona.rudkowska@fsaa.ulaval.ca (I.R.); ann-marie.paradis@fsaa.ulaval.ca (A.-M.P.); elisabeth.thifault@fsaa.ulaval.ca (E.T.); veronique.garneau@fsaa.ulaval.ca (V.G.); simone.lemieux@fsaa.ulaval.ca (S.L.); patrick.couture@crchul.ulaval.ca (P.C.); 2 Endocrinology and Genomics, Laval University Medical Center, Quebec G1V 0A6, Canada

**Keywords:** triacylglycerol, metabolic pathways, lipids, genotypes, *FADS* gene cluster, polyunsaturated fatty acid omega-3

## Abstract

Eicosapentaenoic and docosahexaenoic acids have been reported to have a variety of beneficial effects on cardiovascular disease risk factors. However, a large inter-individual variability in the plasma lipid response to an omega-3 (*n*-3) polyunsaturated fatty acid (PUFA) supplementation is observed in different studies. Genetic variations may influence plasma lipid responsiveness. The aim of the present study was to examine the effects of a supplementation with *n*-3 PUFA on the plasma lipid profile in relation to the presence of single-nucleotide polymorphisms (SNPs) in the fatty acid desaturase (*FADS*) gene cluster. A total of 208 subjects from Quebec City area were supplemented with 3 g/day of *n*-3 PUFA, during six weeks. In a statistical model including the effect of the genotype, the supplementation and the genotype by supplementation interaction, SNP rs174546 was significantly associated (*p* = 0.02) with plasma triglyceride (TG) levels, pre- and post-supplementation. The *n*-3 supplementation had an independent effect on plasma TG levels and no significant genotype by supplementation interaction effects were observed. In summary, our data support the notion that the *FADS* gene cluster is a major determinant of plasma TG levels. SNP rs174546 may be an important SNP associated with plasma TG levels and *FADS*1 gene expression independently of a nutritional intervention with *n*-3 PUFA.

## 1. Introduction

High triacylglycerol (TG) levels are associated with cardiovascular disease (CVD) [1]. Population mean TG levels have increased since 1976 in parallel with the constant growing epidemic of obesity, insulin resistance and type-2 diabetes mellitus [2,3]. A meta-analysis of 17 population-based prospective trials including 46,413 men and 10,864 women identified plasma TG levels as an independent risk factor of CVD and estimated that for each increase of 1.0 mmol/L, the relative risk of CVD increased approximately by 30% for men and 75% for women [4,5]. 

Numerous studies have demonstrated the beneficial effects of omega-3 (*n*-3) polyunsaturated fatty acids (PUFA), especially eicosapentaenoic acid (EPA) and docosahexaenoic acid (DHA) on reducing CVD risk factors [6,7,8,9]. The intake of EPA and DHA has been associated with a reduced risk of myocardial infarction and the prevalence of recurrence [10,11]. A review of human studies reported that 4 g of marine-derived *n*-3 PUFA per day decreased plasma TG concentrations by 25% to 30% [4]. The American Heart Association recommends an intake of 2 to 4 g of EPA/DHA per day for patients who need to lower their plasma TG levels [12]. The decreased in plasma TG that is observed with high intakes of *n*-3 PUFA appears to be secondary to the increased hepatic β-oxidation and decreased lipogenesis [13].

The Fish Oil Intervention and Genotype (FINGEN) study showed that TG levels of 31% of the volunteers did not show any reduction after an 8-week supplementation with 1.8 g/day of EPA + DHA [14]. Innate characteristics such as gender, age and genetic factors may contribute to the variability in benefits reported from intervention trials using *n*-3 PUFAs [15]. Therefore, the anticipated effect of such a supplementation on an individual basis does not necessarily match those reported for the general population [16]. 

The large inter-individual variability observed in the plasma lipid response to a supplementation with *n*-3 PUFA may partly result from genetic variations. Recent data suggest that single-nucleotide polymorphisms (SNPs) found in genes involved in metabolic pathways of *n*-3 PUFA contribute to the variability of PUFA levels [15,17]. The fatty acid desaturase-1 (*FADS*1) and fatty acid desaturase-2 (*FADS*2) genes encode respectively for two desaturases: δ-5 desaturase (D5D) and δ-6 desaturase (D6D) [17]. The D5D and D6D, responsible for double bonds formation in the *n*-3 PUFA pathways, have been associated with differences in fatty acid (FA) composition of plasma [18], erythrocyte membranes [19] and adipose tissue [18]. Another potential desaturase, whose function remains to be elucidated, is possibly encoded by the fatty acid desaturase-3 gene (*FADS*3) [20].

Concerns regarding the efficiency of *n*-3 PUFA supplementation remain. The TG lowering effects of *n*-3 PUFA using several ratios and doses of EPA and DHA have been reported in different studies. However, these studies do not allow setting the optimal conditions. Conflicting data exist and may arise from inter-individual genetic variations. The purpose of the present study is to test whether the plasma lipid response to a 6-week n-3 PUFA supplementation is influenced by genetic variations in the *FADS* gene cluster.

## 2. Subjects and Methods

### 2.1. Study Population

A total of 254 subjects from the greater Quebec City metropolitan area were recruited between September 2009 and December 2011 through advertisements in local newspapers as well as by electronic messages sent to university students/employees. However, only 208 subjects were eligible for further analyses. Missing values of blood lipid profile pre- and/or post-supplementation did not allow those 46 subjects to be included in statistical analyses. Participants were aged between 18 and 50 years. They were non-smokers, with a body mass index (BMI) between 25 and 40 kg/m^2^ and with no current lipid-lowering medications. Subjects were not included if they had taken *n*-3 PUFA supplements for at least 6 months prior, used oral hypolipidemic therapy or had been diagnosed with diabetes, hypertension, hypothyroidism or other known metabolic disorders such as severe dyslipidemia or coronary heart disease. The experimental protocol was approved by the ethics committees of Laval University Hospital Research Center and Laval University. This trial was registered at clinicaltrials.gov as NCT01343342. 

### 2.2. Study Design and Diets

Two hundred and eight subjects followed a run-in period of two weeks where a trained registered dietitian gave individual dietary instructions. Recommendations were drawn from the Canada’s Food Guide to Healthy Eating. All subjects were asked to apply these dietary recommendations and to maintain stable body weight throughout the protocol. Among these recommendations, some specifications have been imposed to ensure the success of this study such as not to exceed two portions of fish or seafood per week (maximum 150 g) and to choose, preferably, fish other than oily fish known to be richer in *n*-3 PUFA as fish with white flesh. With the growing popularity of grocery products fortified with *n*-3 PUFA, participants were asked to avoid these products during the study period. Among these enriched products, some eggs, milk, juice, bread and yogurt have been identified. Subjects were also asked to limit their alcohol intakes to no more than two drinks per week. Subjects were not allowed to take *n*-3 PUFA supplements, including those of vegetable sources, and to take vitamins or natural health products during the protocol. 

After the run-in period, each participant received a bottle containing *n*-3 PUFA capsules (Ocean Nutrition, Nova Scotia, Canada) covering the following 6-week period. They had to take 5 capsules per day, which gave them a total of 3 g of *n*-3 PUFA (1.9 g EPA and 1.1 g DHA) per day. Compliance was measured by bottles returning and by calculating the number of remaining capsules in the bottles at the end of the supplementation. Subjects had to report any deviations that may have occurred during the protocol. They also had to write their alcohol and fish consumption on a log sheet. Before each phase of the study, subjects received written and oral dietary instructions by a registered dietitian.

A dietitian administrated a validated food-frequency questionnaire (FFQ) before the run-in period to each participant [21]. This FFQ is based on typical food items available in the province of Quebec and contains a total of 91 items; 27 items had between 1 and 3 subquestions. The subjects were asked how often they consumed each item per day, per week, per month, or none at all during the last month. Many examples of portion size were provided for a better estimation of the real portion consumed by the subject. Dietary intake data were analyzed using Nutrition Data System for Research software version 2011 developed by the Nutrition Coordinating Center (NCC), University of Minnesota, Minneapolis, MN.

## 3. Anthropometric Measurements

Body weight, height, and waist girth were measured according to the procedures recommended by the Airlie Conference [22] and were taken before the run-in period, as well as pre- and post-*n*-3 PUFA supplementation. BMI was calculated as weight in kilograms divided by height in meters squared (kg/m^2^).

## 4. Biochemical Parameters

Blood samples were collected from an antecubital vein into vacutainer tubes containing EDTA after 12 h overnight fast and 48 h alcohol abstinence. Blood samples were taken to identify and exclude individuals with any metabolic disorders. Afterwards, participants had blood samples taken prior to and after the *n*-3 PUFA supplementation period. Plasma was separated by centrifugation (2500× *g* for 10 min at 4 °C) and samples were aliquoted and frozen for subsequent analyses. Plasma total cholesterol (TC) and TG concentrations were measured using enzymatic assays [23]. The high-density lipoprotein cholesterol (HDL-C) fraction was obtained after precipitation of very low-density lipoprotein and low-density lipoprotein particles in the infranatant with heparin manganese chloride [24]. Low-density lipoprotein cholesterol (LDL-C) was calculated with the Friedewald formula [25]. Apolipoprotein B-100 (ApoB100) concentrations were measured in plasma by the rocket immunoelectrophoretic method of Laurell, as previously described [26]. Plasma C-reactive protein (CRP) was measured by nephelometry (Prospec equipment Behring) using a sensitive assay, as described previously [27]. 

### 4.1. SNP Selection and Genotyping

SNPs in *FADS*1, *FADS*2 and *FADS*3 were identified using the International HapMap Project SNP database, based on the National Center for Biotechnology Information (NCBI) B36 assembly Data Rel 28 phase II + III, build 126 (Table 1). Tagger procedure in Haploview V4.2 was used to determine tag SNPs (tSNPs) using a minor allele frequency (MAF) >1% and pairwise tagging (*R*^2^ ≥ 0.8). Subsequently, we examined linkage disequilibrium (LD) out of the 19 SNPs covering all common variations in the *FADS* gene cluster area, using the LD Plot procedure in Haploview V4.2. Most of the SNPs were in LD (*R*^2^ ≥ 0.8) and the mean *R*^2^ was 0.953, so 19 SNPs were sufficient to cover the entire area. The SIGMA GenElute Gel Extraction Kit (Sigma-Aldrich Co., St. Louis, MO, USA) has been used to extract genomic DNA. Selected SNPs of the *FADS* gene cluster (rs174456, rs174627, rs482548, rs2072114, rs12807005, rs174448, rs2845573, rs7394871, rs7942717, rs74823126, rs174602, rs498793, rs7935946, rs174546, rs174570, rs174579, rs174611, rs174616 and rs968567) have been genotyped using validated primers and TaqMan probes (Applied Biosystems, Foster City, CA, USA) [28]. DNA was mixed with TaqMan Universal PCR Master Mix (Applied Biosystems), with a gene-specific primer and with probe mixture (predeveloped TaqMan SNP Genotyping Assays; Applied Biosystems) in a final volume of 10 μL. Genotypes were determined using a 7500 RT-PCR System and analyzed using ABI Prism SDS version 2.0.5 (Applied Biosystems, Foster City, CA, USA).

**Table 1:** Selected polymorphisms in the fatty acid desaturase (*FADS*)gene cluster.

Gene	dbSNP No. ^1^	Sequence ^2^	Position	MAF	Genotype/Frequency		
*FADS*3	rs174456	CTACTAC[A/C]TGGCAGC	intron	0.299	A/A (*n* = 102)	A/C (*n* = 89)	C/C (*n* = 18)
					0.488	0.426	0.086
Intergenic	rs174627	TTATCTG[C/T]GTAGCTA	Intergenic	0.124	A/A (*n* = 2)	A/G (*n* = 48)	G/G (*n* = 159)
*FADS* 2-					0.010	0.230	0.761
*FADS* 3							
*FADS*2	rs482548	GGGACAC[C/T]GTGGGGA	3′ UTR	0.126	C/C (*n* = 161)	C/T (*n* = 40)	T/T (*n* = 6)
					0.778	0.193	0.029
*FADS*2	rs2072114	AGAGTTC[A/G]GGTCTTA	Intron	0.110	A/A (*n* = 167)	A/G (n = 38)	G/G (*n* = 4)
					0.799	0.182	0.019
Intergenic	rs12807005	GATCATG[A/G]ATCACTG	Intergenic	0.012	A/A (*n* = 0)	A/G (*n* = 5)	G/G (n = 204)
*FADS* 1-							
*FADS* 2					0.000	0.024	0.976
Intergenic **	rs174448	ACCCTGA[C/T]TTCTGGG	Intergenic	0.363	A/A (*n* = 78)	A/G (*n* = 109)	G/G (*n* = 21)
*FADS* 2- **							
*FADS* 3					0.375	0.524	0.101
*FADS*2	rs2845573	TTGCTCA[C/T]GTTACTC	Intron	0.081	A/A (*n* = 177)	A/G (*n* = 30)	G/G (*n* = 2)
					0.847	0.144	0.010
*FADS*3	rs7394871	AAGGGAC[A/C]CCTGCCC	Intron	0.072	A/A (*n* = 2)	A/C (*n* = 26)	C/C (*n* = 181)
					0.010	0.124	0.866
*FADS*3	rs7942717	CCAAACG[A/G]GTGCCTG	Intron	0.117	A/A (*n* = 161)	A/G (*n* = 47)	G/G (*n* = 1)
					0.770	0.225	0.005
Intergenic **	rs7482316	TTTTCAA[A/G]CTGCCGA	Intergenic	0.103	A/A (*n* = 168)	A/G (*n* = 39)	G/G (*n* = 2)
*FADS* 2- **							
*FADS* 3					0.804	0.187	0.010
*FADS*2	rs174602	CCAACCC[A/G]TCCTGC	Intron	0.184	C/C (*n* = 9)	C/T (*n* = 59)	T/T (*n* = 141)
					0.043	0.282	0.675
*FADS*2	rs498793	CTGTAAC[A/G]CAGGCTG	Intron	0.456	C/C (*n* = 62)	C/T (*n* = 99)	T/T (*n* = 43)
					0.098	0.717	0.186
*FADS*2	rs7935946	AAGGTTC[C/T]GGGAACT	Intron	0.041	C/C (*n* = 195)	C/T (*n* = 11)	T/T (*n* = 3)
					0.933	0.053	0.014
*FADS*1	rs174546	CCTCTGC[C/T]TTGGCTC	3′ UTR	0.297	C/C (*n* = 103)	C/T (*n* = 86)	T/T (*n* = 19)
					0.498	0.412	0.091
*FADS*2	rs174570	AACTTGA[C/T]GTAGATC	Intron	0.125	C/C (*n* = 159)	C/T (*n* = 46)	T/T (*n* = 3)
					0.764	0.221	0.014
*FADS*2	rs174579	TCCCTTT[C/T]CAGGAAG	Intron	0.202	C/C (*n* = 127)	C/T (*n* = 78)	T/T (*n* = 3)
					0.611	0.375	0.014
*FADS*2	rs174611	TCCTGGA[C/T]CCTGAGA	Intron	0.258	C/C (*n* = 12)	C/T (*n* = 84)	T/T (*n* = 113)
					0.057	0.402	0.541
*FADS*2	rs174616	GACCTCA[C/T]GTTCCAA	Intron	0.498	A/A (*n* = 51)	A/G (*n* = 108)	G/G (*n* = 50)
					0.244	0.517	0.239
*FADS*2	rs968567	TCCCCGG[A/G]AGCTCAG	5′ UTR	0.160	A/A (*n* = 2)	A/G (*n* = 63)	G/G (*n* = 144)
					0.010	0.301	0.689

^1^ dbSNP No. from HapMap Data Rel 28 Phase II + III, August 10 on NCBI b36 Assembly dbSNP b126 database; ^2^ Genes sequences from dbSNP short genetics variations NCBI reference assembly.

### 4.2. Gene Expression of the *FADS* Gene Cluster

cDNA was mixed with TaqMan Universal PCR Master Mix (Applied Biosystems) and a gene-specific primer and probe mixture (predeveloped TaqMan Gene Expression Assays; Applied Biosystems) in a final volume of 20 μL. The assays used were as follows: Hs00203685_ml (*FADS*1), Hs00188654_ml (*FADS*2), Hs00222230_ml (*FADS*3) and Hs99999905_ml (glyceraldehyde-3-phosphate dehydrogenase (GADPH)) as the housekeeping gene. Assays used the same fluorescent reporter probe (FAM™ dye-labeled) and thus each combination treatment and gene was analyzed in individual wells on a 96-well plate. All samples were run in duplicate on an Applied Biosystems 7500 Fast Real Time PCR System (Applied Biosystems) using the following thermal cycling profile: 50 °C (2 min), 95 °C (10 min), followed by 40 steps of 95 °C for 15 s and 60 °C for 60 s. The RT-PCR results were imported into ExpressionSuite Software v1.0 (Life Technologies). Data were adjusted for the endogenous control (GADPH). 

## 5. Statistical Analyses

Data were analyzed with SAS statistical software V9.2 (SAS Institute, Cary, NC, USA) The ALLELE procedure was used to verify the departure from Hardy-Weinberg equilibrium (HWE) and to calculate minor allele frequency (MAF). Variables not normally distributed were log-transformed before analyses. ANOVA and the type III sum of squares were used to look for significant differences in daily energy and nutrient intakes, at prior and after an *n*-3 PUFA supplementation when age, sex and BMI were included in the model and to test for differences in plasma TG levels among groups divided on the basis of the genotype for rs174546. The repeated MIXED procedure was used to test for the effects of the genotype, the supplementation and the genotype by supplementation interaction on plasma TG and gene expression levels when age, sex and BMI were included in the model. Statistical significance was defined as *p* ≤ 0.05. To identify potential effects of variations located in the *FADS* gene cluster region, a transcription factor search was performed using MatInspector 8.0 software from the Genomatix Suite. 

## 6. Results

**Figure 1:**
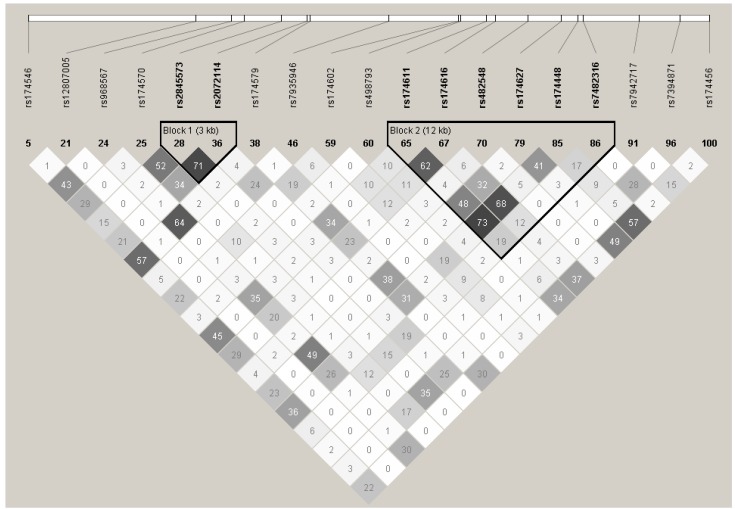
Linkage disequilibrium (LD) plot of SNPs of the fatty acid desaturase (*FADS*)gene cluster.

All SNPs were in HWE except two: rs7935946 and rs174579 (see Figure 1 for the LD plot). These SNPs were not considered for further analysis. Therefore, associations with 17 SNPs were tested in statistical analyses. The % gene coverage with these 17 SNPs was of 87%.

Baseline characteristics of study participants are presented in Table 2. According to these results, men and women were overweight (BMI > 25 kg/m^2^) and had mean plasma TG levels slightly above the cut-point value of 1.129 mmol/L recommended by the AHA for optimal plasma TG levels [29]. Gender differences are evident with respect to weight, TC/HDL-C ratio, CRP, HDL-C and TG levels.

**Table 2:** General characteristics of the study sample before *n*-3 PUFA supplementation.

		All ^1^	Men ^1^	Women ^1^	*p* Values
Population: Men/Women		208	96	112	
Age (years)		30.8 ± 8.7	31.2 ± 8.1	30.5 ± 9.1	0.55
Weight (kg) ^3^		81.4 ± 13.9	87.2 ± 13.4	76.4 ± 12.3	<0.0001
BMI (kg/m^2^) ^2^^,^^3^		27.8 ± 3.7	27.5 ± 3.6	28.2 ± 3.8	0.13
Waist circumference (cm) ^3^		93.3 ± 10.8	94.8 ± 11.0	92.0 ± 10.4	0.06
Cholesterol (mM) ^4^					
	Total	4.82 ± 1.00	4.80 ± 1.00	4.83 ± 1.02	0.75
	HDL	1.46 ± 0.39	1.29 ± 0.31	1.61 ± 0.39	<0.0001
	LDL	2.79 ± 0.87	2.91 ± 0.87	2.69 ± 0.86	0.08
Total chol./HDL ratio ^4^		3.49 ± 1.04	3.91 ± 1.13	3.12 ± 0.80	<0.0001
Triacylglycerols (mM) ^2^^,^^4^		1.23 ± 0.64	1.32 ± 0.74	1.15 ± 0.53	0.04
ApoB100 (g/L) ^4^		0.86 ± 0.25	0.89 ± 0.25	0.84 ± 0.25	0.12
CRP (mg/L) ^2^^,^^4^		3.13 ± 7.10	1.66 ± 2.45	4.39 ± 9.24	0.02

^1^ Values are means ± SD; ^2^*p*-Value derived from log_10_-transformed; ^3^ Results were adjusted for age; ^4^ Results were adjusted for age and BMI.

Daily energy intakes were calculated by a food frequency questionnaire validated for healthy French-Canadian men and women [21] and are presented in Table 3. After the supplementation, carbohydrates, saturated fats and proteins were significantly different from the pre *n*-3 PUFA period (*p* = 0.0005, *p* = 0.0008 and *p* = 0.02, respectively). After the supplementation, the PUFA intake—taking into account food intakes and fish oil capsules used during the supplementation—was significantly increased (*p* = 0.003). In our study, the 6-week average of fish servings/week (a serving = 75 g of fish) was 0.89 servings/week based on the compliance questionnaire given at the end of the study. Furthermore, subjects were asked to limit their fish consumption to no more than 2 servings/week (maximum of 150 g). Based on these results, subjects who had consumed the maximum quantity of fish permitted per week would have had an extra 0.43 g of EPA + DHA/day. With the fish oil supplementation, the total of EPA + DHA was 3 g/day. Common food items eaten by the subjects were seafood, tuna, trout, haddock and salmon. 

**Table 3:** Daily intakes from food frequency questionnaire (*n*=208).

Nutrients	Pre-*n*-3 PUFA	Post-*n*-3 PUFA	*p*
Energy, Kcal	2272 ± 590	2143 ± 566	0.08
Total lipids, g	86.5 ± 29.2	86.6 ± 29.8	0.48
MUFA, g	30.8 ± 11.8	29.6 ± 12.4	0.13
PUFA, g	15.2 ± 6.6	17.1 ± 6.9	0.003
SFA, g	29.0 ± 12.0	25.5 ± 10.4	0.0008
Cholesterol, mg	303.7 ± 147.4	297.3 ± 169.4	0.41
Carbohydrates, g	286.7 ± 78.9	263.4 ± 77.7	0.0005
Protein, g	97.8 ± 30.2	92.6 ± 29.6	0.02
Alcohol, g	3.2 ± 6.0	3.2 ± 6.1	0.81

All values are mean ± SD; MUFA = monounsaturated fatty acids; PUFA = polyunsaturated fatty acids; SFA = saturated fatty acids; All date were adjusted for sex, age and BMI in ANCOVA; *p* Values for dietary intakes between pre- and post-supplementation are calculated using ANOVA; Statistical significance was defined as *p* ≤ 0.05.

**Figure 2:**
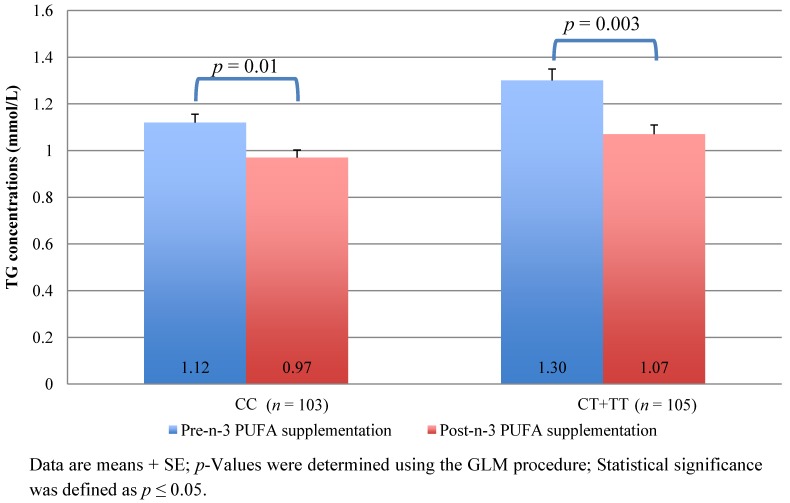
TG concentrations before and after a 6-week *n*-3 PUFA supplementation according to the SNP rs174546 in *FADS*1.

To test the potential interaction between the *FADS* gene polymorphisms and the *n*-3 PUFA supplementation on plasma TG levels, the MIXED procedure was used in order to test whether the genotype, the supplementation or the interaction (genotype by supplementation) were associated with plasma TG levels. As shown in Table 4, independently of the genotype, the supplementation was associated with fasting plasma TG concentrations meaning that the supplementation had an independent effect on plasma TG levels, as expected. One SNP was associated with plasma TG concentrations, rs174546, suggesting that this polymorphism modulates plasma TG levels. No significant genotype by supplementation interaction effects were observed. Further analyses revealed that, in the pre-supplementation period, plasma TG levels were lower in CC homozygotes when compared to carriers of the minor T allele (Table 5, *p* = 0.002). In the post-supplementation period, both genotype groups significantly decreased their plasma TG levels (Figure 2). However, there was no significant difference in post-supplementation plasma TG levels between the genotype groups when age, sex and BMI were included in the model (Table 5). Results remained unchanged after further adjustment for pre-supplementation plasma TG levels (data not shown). Since there was no significant difference between the two groups in post-supplementation plasma TG levels and since the interaction term was not significant in the repeated model, these results suggest that the *FADS* rs174546 is associated with plasma TG levels only and not with the plasma TG response to the *n*-3 PUFA supplementation. 

**Table 4:** Effect of the genotype, the *n*-3 PUFA supplementation and the interaction (genotype by supplementation) on TG levels (*n*=208).

SNPs	Genotype **	Supplementation **	Interaction **
	*p*	*p*	*p*
	β	β	β
rs174456	0.77	0.0001 *	0.67
	0.0013 ± 0.027	0.081 ± 0.027	−0.016 ± 0.038
rs174627	0.23	0.0002 *	0.51
	−0.013 ± 0.031	0.094 ± 0.038	−0.027 ± 0.044
rs482548	0.79	0.0001 *	0.48
	−0.023 ± 0.032	0.048 ± 0.039	0.032 ± 0.045
rs2072114	0.85	0.002 *	0.99
	0.0046 ± 0.034	0.073 ± 0.042	−0.00012 ± 0.047
rs12807005	0.06	0.35	0.68
	−0.13 ± 0.080	0.027 ± 0.11	0.048 ± 0.11
rs174448	0.22	0.0003 *	0.49
	−0.010 ± 0.028	0.083 ± 0.024	−0.027 ± 0.039
rs2845573	0.61	0.01 *	0.57
	−0.028 ± 0.037	0.049 ± 0.048	0.020 ± 0.052
rs7394871	0.46	0.009 *	0.87
	0.017 ± 0.039	0.066 ± 0.051	0.0082 ± 0.055
rs7942717	0.56	0.0007 *	0.9
	0.014 ± 0.032	0.078 ± 0.039	−0.0057 ± 0.045
rs7482316	0.69	0.002 *	0.99
	−0.0070 ± 0.033	0.074 ± 0.042	−0.0013 ± 0.047
rs174602	0.8	0.0001 *	0.5
	0.017 ± 0.029	0.091 ± 0.033	−0.026 ± 0.041
rs498793	0.83	0.01*	0.78
	−0.029 ± 0.029	0.071 ± 0.023	0.0080 ± 0.042
rs174546	0.02 *	<0.0001 *	0.55
	−0.035 ± 0.027	0.084 ± 0.026	−0.023 ± 0.038
rs174570	0.58	0.001 *	0.64
	−0.022 ± 0.032	0.058 ± 0.039	0.020 ± 0.044
rs174611	0.09	<0.0001 *	0.7
	−0.025 ± 0.027	0.081 ± 0.028	−0.014 ± 0.038
rs174616	0.37	0.0005 *	0.84
	−0.022 ± 0.031	0.071 ± 0.022	0.0073 ± 0.044
rs968567	0.13	0.0001 *	0.54
	−0.019 ± 0.029	0.090 ± 0.033	−0.024 ± 0.041

*p*-Values are derived from log transformed data; β represents the effect size + SE; All results were adjusted for BMI, age and sex; The MIXED models implemented in SAS version 9.2 were used to test interaction effects.

**Table 5:** Triacylglycerol (TG) concentrations according to genotype distributions of the *FADS* gene cluster polymorphisms before and after a 6-week *n*-3 PUFA supplementation.

SNPs		Pre- *n*-3 PUFA supplementation			Post- *n*-3 PUFA supplementation		
		11	12 + 22	*p*	11	12 + 22	*p*
rs174456	Genotype	AA (*n* = 102)	AC + CC (*n* = 106)		AA (*n* = 102)	AC + CC (*n* = 106)	
	TG levels	1.19 ± 0.61	1.23±0.65	0.45	1.03±0.58	1.01±0.47	0.96
rs174627	Genotype	CC (*n* = 159)	AC + AA (*n* = 49)		CC (*n* = 159)	AC + AA (*n* = 49)	
	TG levels	1.18±0.58	1.31±0.74	0.06	1.01±0.47	1.05±0.66	0.55
rs482548	Genotype	CC (*n* = 161)	CT + TT (*n* = 46)		CC (*n* = 161)	CT+TT (*n* = 46)	
	TG levels	1.22±0.64	1.17±0.58	0.62	1.02±0.53	1.03±0.50	0.29
rs2072114	Genotype	AA (*n* = 166)	AG + GG (*n* = 42)		AA (*n* = 166)	AG + GG (*n* = 42)	
	TG levels	1.20±0.64	1.22±0.59	0.83	1.02±0.55	1.03±0.42	0.87
rs12807005	Genotype	CC (*n* = 204)	AC + AA (*n* = 4)		CC (*n* = 204)	AC + AA (*n* = 4)	
	TG levels	1.21±0.63	1.26*±* 0.52	0.15	1.01±0.52	1.21±0.53	0.02 *
rs174448	Genotype	AA (*n* = 78)	AG + GG (*n* = 130)		AA (*n* = 78)	AG + GG (*n* = 130)	
	TG levels	1.14±0.57	1.25±0.66	0.06	0.99±0.50	1.04±0.54	0.59
rs2845573	Genotype	AA (*n* = 176)	AG + GG (*n* = 32)		AA (*n* = 176)	AG + GG (*n* = 32)	
	TG levels	1.22±0.67	1.15±0.36	0.98	1.02±0.54	1.05±0.39	0.30
rs7394871	Genotype	CC (*n* = 180)	AC + AA (*n* = 28)		CC (*n* = 180)	AC + AA (*n* = 28)	
	TG levels	1.22±0.65	1.13±0.42	0.37	1.03±0.54	0.98±0.40	0.53
rs7942717	Genotype	AA (*n* = 160)	AG + GG (*n* = 48)		AA (*n* = 160)	AG + GG (*n* = 48)	
	TG levels	1.20±0.59	1.25±0.73	0.64	1.02±0.52	1.04±0.53	0.61
rs7482316	Genotype	AA (*n* = 167)	AG + GG (*n* = 41)		AA (*n* = 167)	AG + GG (*n* = 41)	
	TG levels	1.19±0.58	1.28±0.79	0.73	1.01±0.52	1.07±0.55	0.77
rs174602	Genotype	TT (*n* = 140)	CT + TT (*n* = 68)		TT (*n* = 140)	CT + TT (*n* = 68)	
	TG levels	1.19±0.61	1.24±0.67	0.65	1.03±0.56	0.99±0.44	0.42
rs498793	Genotype	CC (*n* = 62)	CT + TT (*n* = 142)		CC (*n* = 62)	CT + TT (*n* = 142)	
	TG levels	1.19±0.67	1.21±0.61	0.30	0.99±0.54	1.03±0.52	0.17
rs174546	Genotype	CC (*n* = 103)	CT + TT (*n* = 105)		CC (*n* = 103)	CT + TT (*n* = 105)	
	TG levels	1.12±0.51	1.30±0.71	0.002 *	0.97±0.46	1.07±0.58	0.07
rs174570	Genotype	CC (*n* = 159)	CT + TT (*n* = 49)		CC (*n* = 159)	CT + TT (*n* = 49)	
	TG levels	1.21±0.65	1.19±0.54	0.91	1.01±0.54	1.06±0.46	0.33
rs174611	Genotype	TT (*n* = 113)	CT + CC (*n* = 95)		TT (*n* = 113)	CT + CC (*n* = 95)	
	TG levels	1.14±0.48	1.29±0.75	0.04 *	0.99±0.45	1.06±0.60	0.19
rs174616	Genotype	AA (*n* = 51)	AG + GG (*n* = 157)		AA (*n* = 51)	AG + GG (*n* = 157)	
	TG levels	1.24±0.73	1.20±0.59	0.48	1.00±0.53	1.03±0.52	0.32
rs968567	Genotype	GG (*n* = 143)	AG + GG (*n* = 65)		GG (*n* = 143)	AG + GG (*n* = 65)	
	TG levels	1.17±0.58	1.30±0.71	0.03 *	1.00±0.47	1.06±0.62	0.36

Data are TG levels ± SD; *p*-Values are adjusted for age, sex and BMI; 11 stands for major allele homozygote carriers; 12 + 22 stand for minor allele carriers (homozygotes and heterozygotes); statistical significance was defined as *p* ≤ 0.05.

In a model testing the effect of the genotype, the supplementation or the interaction (genotype by supplementation) on *FADS*1 gene expression, the SNP rs174546 was associated with *FADS*1 gene expression (*p* = 0.01) after adjustments for age, sex and BMI (data not shown). No effect of the *n*-3 PUFA supplementation (*p* = 0.54) and no gene by supplementation effect (*p* = 0.56) explained *FADS*1 gene expression. 

## 7. Discussion

In this study, we tested whether plasma TG levels during an *n*-3 PUFA supplementation varied according to the presence of common polymorphisms in the *FADS* gene cluster. 19 SNPs were initially chosen from the *FADS* gene cluster area covering all common variations of the *FADS* gene cluster. After genotyping, 17 SNPs were in HWE and thus were analysed in the present study. The *FADS* gene cluster area has been chosen due to the role of D5D and D6D activity in the *n*-3 PUFA metabolic pathway. D5D and D6D are essential parts of PUFA biosynthesis that catalyze a series of desaturation processes [30]. These desaturases are respectively encoded by *FADS*1 and *FADS*2. Also, a Genome-Wide Association Study has shown that the strongest evidence for an association of genetic contributors of plasma PUFA concentrations was observed in the *FADS* gene cluster area [31]. Some polymorphisms come from intergenic regions (rs174627, rs12807005. rs174448 and rs7482316) and are part of the *FADS*1 and *FADS*2 gene promoters because of the head to head orientation. *FADS*3 promoter was not considered since no desaturase activity is reported (see all selected SNPs in Table 1). All SNPs were polymorphic for the selected study population and were not in strong LD with each other. 

In the present study, we observed an independent genotype effect of the SNP rs174546 on plasma TG levels and on *FADS*1 gene expression levels in a model including the SNP, the supplementation effect and the SNP by supplementation interaction. In the literature, SNP rs174546 has been much studied. Numerous studies have attributed beneficial effects to this polymorphism. Indeed. Dumont *et al.* showed that the minor allele of rs174546 was associated with decreased plasma TC and non-HDL-C levels [32]. In another study, Lu *et al.* reported similar results where the common C allele was associated with higher levels of TC non-HDL-C and HDL-C levels, but only in individuals consuming high intakes of omega-6 (>5.26% of total energy intake) [33]. Another study demonstrated that rs174547, in strong-LD with rs174546 (*R^2^* = 1.0), was a dominant SNP in the *FADS* gene cluster that influences desaturase activity and some evidence emerging from human-based research demonstrates that genetic variation in human desaturase genes affects enzyme activity and, consequently, disease risk factors [34]. They also showed that homozygotes for the major allele had a higher estimate of aggregate desaturase activity (ADA) reflected in *n*-3 PUFA while homozygotes for the minor allele had the lowest ADA [34]. This SNP is also associated with altered desaturase activity reflected in *n*-6 PUFA [34]. Recent studies have also shown that two common and very distinct FADS haplotypes were strongly associated with long-chain PUFAs synthesis levels. Haplotypes A and D, which includes rs174546, may exert differences in transcription levels and the ability to synthesize essential omega-3 and omega-6 long-chain PUFAs [35]. Reduced substrate (*i.e.*, FA) availability leads to a reduction of VLDL TG synthesis [13]. *FADS* gene cluster, especially rs174546, is associated with TG levels and also to ADA, making it a significant SNP when talking about associations between lipids and FA metabolism.

Some SNPs may modulate desaturase activity and lead to changes in *n*-3 PUFA metabolism. We tested SNP rs174546 for potential functional significance using MatInspector 8.0 software, but it did not seem to alter transcription factor binding sites. 

After the *n*-3 PUFA supplementation, considerable inter-individual variability in plasma TG levels was observed. It appears that some individuals require higher doses to achieve demonstrable benefits, whereas others are highly sensitive to relatively low doses and individuals with certain genotypes may experience adverse responses with respect to specific risk biomarkers, at least at high doses of *n*-3 PUFA [36]. *FADS* gene expression may modulate specific risk biomarkers in relation to certain genotype. Overall, the *n*-3 PUFA supplementation had no effect on *FADS* gene expression. The SNP rs174546 was a significant predictor of *FADS*1 gene expression levels. Caslake *et al*. showed that 31% of all volunteers had no reduction in plasma TG levels after 1.8 g/day of EPA + DHA for 8 weeks [14]. We basically observe similar results in the present study, as 28.8% of the participants had no reduction in plasma TG levels after the 6-week *n*-3 PUFA supplementation. These results demonstrate that intra-individual variability of plasma lipid levels is an important potential source of error enhancing the importance of genetic testing to identify individuals that are more likely to benefit from such therapies [37].

This study presents some limitations. Regarding daily intakes, significant differences for carbohydrates, saturated fats or proteins could be due to recall bias from subjects. Since subjects were asked to follow recommendations drawn from the *Canada’s Food Guide to Healthy Eating*, they could have reported food consumption differences that slightly changed calculated intakes. However, those differences did not affect significantly BMI nor energy intakes. Because carbohydrates intakes were significantly decreased in the post-supplementation period and this could impact on plasma TG levels, the carbohydrate intakes have been added into the statistical model and results remained unchanged (data not shown).

## 8. Conclusions

In summary, our data support the notion that the *FADS* gene cluster, especially SNPs from *FADS*1, are major determinants of plasma TG levels. SNP rs174546 may be an important SNP in the *FADS* gene cluster associated with plasma TG levels and *FADS*1 gene expression independently of a nutritional intervention with *n*-3 PUFA. These results need to be replicated in other independent studies. A better understanding of the phenomenon could allow the development of personalized dietary advice for prevention of CVD. 
